# Attitudes and Values of US Adults Not Yet Up-to-Date on COVID-19 Vaccines in September 2022

**DOI:** 10.3390/jcm12123932

**Published:** 2023-06-08

**Authors:** Matthew Z. Dudley, Holly B. Schuh, Jana Shaw, Daniel A. Salmon

**Affiliations:** 1 Institute for Vaccine Safety, Johns Hopkins Bloomberg School of Public Health, Baltimore, MD 21205, USA; hschuh1@jhu.edu (H.B.S.); dsalmon1@jhu.edu (D.A.S.); 2 Department of International Health, Johns Hopkins Bloomberg School of Public Health, Baltimore, MD 21205, USA; 3 Department of Epidemiology, Johns Hopkins Bloomberg School of Public Health, Baltimore, MD 21205, USA; 4 Department of Pediatrics, SUNY Upstate Medical University, Syracuse, NY 13210, USA; shawja@upstate.edu; 5 Department of Health, Behavior and Society, Johns Hopkins Bloomberg School of Public Health, Baltimore, MD 21205, USA

**Keywords:** COVID-19, vaccine, vaccination, vaccine hesitancy

## Abstract

(1) Background: Periodic resurgences in COVID-19 due to more contagious variants highlight the need to increase coverage of booster doses. (2) Methods: Our September 2022 nationally representative survey of US adults measured COVID-19 vaccination status, intentions, attitudes, values, and confidence in information sources. (3) Findings: Although 85% of the weighted sample reported receiving at least one dose of a COVID-19 vaccine, only 63% reported being up-to-date on COVID-19 vaccines (e.g., received a booster dose). Only 12% of those not yet up-to-date indicated they were likely to get up-to-date as soon as possible, whereas 42% were unlikely to ever get up-to-date, and 46% were still uncertain. Most of those not up-to-date on their COVID-19 vaccines were under 45 years of age (58%), without a bachelor’s degree (76%), making under $75,000 annually (53%), and Republican or Independent (82%). Prevalent concerns about COVID-19 vaccines among those uncertain about getting up-to-date included: potential side effects that have not been figured out yet (88%), speed of development (77%), newness (75%), ingredients (69%), drug companies making money (67%), allergic reactions (65%), and experimenting on people (63%). (4) Conclusions: Nearly half of adults not yet up-to-date on COVID-19 vaccines were uncertain about doing so, indicating an opportunity to support their decision-making.

## 1. Introduction

As of March 2023, four vaccines against Coronavirus Disease 2019 (COVID-19) have been authorized by the United States (US) Food and Drug Administration (FDA): two messenger ribonucleic acid (mRNA) vaccines, Pfizer-BioNTech and Moderna, which were given Emergency Use Authorization (EUA) by the FDA in December 2020 [1,2], the Johnson & Johnson (J&J) viral vector vaccine, which was given EUA in February 2021 [3], but is no longer available in the US, due to safety concerns [4], and the Novavax protein subunit vaccine, which was given EUA in July 2022 [5].

COVID-19 cases and deaths declined initially as vaccine supply caught up to demand in the spring of 2021 [6]. However, cases and deaths have periodically surged since then, largely due to the proliferation of the more contagious variants, such as Delta and Omicron [7]. Though vaccines remained effective in protecting against severe disease and death from new variants, their effectiveness was lower as compared to the original strain and waned over time [8,9,10]. Booster doses first received EUA in September 2021 to counteract waning immunity [11], and updated bivalent booster doses received EUA in August 2022 to increase protection against currently circulating strains [12].

The ongoing evolution of both the SARS-CoV-2 virus and our scientific knowledge has necessitated many updates to public health recommendations throughout the pandemic. This has led to public confusion and pandemic fatigue [13,14]. Vaccines have also unfortunately become a polarizing political issue during the pandemic [13]. This threatens public confidence in not only COVID-19 vaccines, but routine vaccines as well. Vaccine hesitancy is complex and influenced by a broad range of factors, such as the compulsory nature of vaccine mandates, coincidental temporal relationships to adverse health events, and lack of trust in government and the pharmaceutical industry [15]. Predictors of hesitancy to receive routine vaccines prior to the pandemic included socioeconomic factors, such as gender, education, income, region of residence, and race/ethnicity [16,17,18], as well as attitudinal constructs, such as confidence in vaccine efficacy and safety, perceived risk of vaccine-preventable diseases, and trust in sources of vaccine information [16]. Regular collection and consideration of representative public survey data is necessary to understand changes and trends in vaccine confidence and concerns.

We initially conducted a nationally representative survey in December 2020 (immediately prior to the first EUA) to measure COVID-19 vaccine intentions, attitudes, and values among US adults [19]. We found that half of US adults intended to likely receive the vaccine once available, while 40% were uncertain and 10% were unlikely to ever vaccinate. Intent to vaccinate (unadjusted) was lower among women, Black Americans, young adults, Republicans, those with lower income, those with larger households, those living in a non-metro area, and those without a bachelor’s degree. Trust in public health authorities was strongly associated with intending to vaccinate against COVID-19.

We conducted additional nationally representative surveys in July [20] and September [21] of 2021 to capture snapshots of COVID-19 vaccine attitudes, values, and intentions at different stages in the pandemic. About three-quarters of US adults reported having received at least one dose of COVID-19 vaccination at this time (74% in July and 77% in September). Of those still unvaccinated as of September 2021, 6% intended to vaccinate, 40% were unlikely to ever vaccinate, and 55% remained uncertain.

Herein, we describe the findings from a fourth nationally representative survey. This survey was conducted in September 2022, which was a year since the last such survey, a year since booster doses were first authorized [11], and immediately following authorization of the updated bivalent boosters [12]. Our main focus was on those not “up-to-date” on their COVID-19 vaccines (e.g., those not yet receiving a booster dose).

## 2. Materials and Methods

### 2.1. Survey Platform

This nationally representative cross-sectional survey was conducted in English and Spanish between 1–12 September 2022, from a new set of respondents randomly identified from the Ipsos KnowledgePanel [22], a probability-based web panel sampling adults (18+ years of age) from all US households that was also used for previous surveys [19,20,21]. To improve the sample’s representativeness of the US population, households without internet access were given internet access and tablet computers. Hispanic Americans were supplementally recruited through random digit dialing in area codes with concentrated Hispanic populations. Enrollment quotas ensured that the sample’s sociodemographic distribution approximated the US, with 50% oversampling of Black and Hispanic respondents. Data from this survey were compared to data from a previous nationally representative survey, which was conducted in July 2021 using the same methodology as described above [20].

This work was considered public health surveillance and, thus, not human subject research by the Institutional Review Board at the Johns Hopkins Bloomberg School of Public Health.

### 2.2. Survey Content

The primary outcomes of this survey were self-reported receipt of COVID-19 vaccines (including boosters) and intentions among those not yet up-to-date. Respondents began the survey by identifying themselves as either (1) up-to-date (i.e., fully vaccinated and boosted), (2) vaccinated, but not up-to-date (e.g., have not gotten booster yet), (3) not having received any COVID-19 vaccines, or (4) prefer not to say (which was treated as missing data for this survey item). Respondents who did not report already being up-to-date then elucidated their intentions to get up-to-date as either (1) definitely soon, (2) probably soon, (3) probably eventually but not right away, (4) only if required, (5) probably not, or (6) definitely not.

This survey measured attitudes about COVID-19 vaccines and disease specific to both adults and children, such as perceived disease susceptibility/severity and the importance of vaccines to control the pandemic. Among those not yet up-to-date and not definitely intending to get up-to-date soon, vaccine concerns and other reasons for not vaccinating were selected. Self-reported influenza vaccination coverage, confidence in sources of information about COVID-19, and cumulative prevalence of COVID-19 disease (e.g., ever having COVID-19) were captured. Trust in the CDC was measured as a construct via a 14-question scale [23]. Sociodemographic characteristics including gender, race/ethnicity, age, education, income, political affiliation, region, and metropolitan statistical area (MSA) were available for all Ipsos KnowledgePanel members. Many of the other items measured in this survey were also measured in the July 2021 survey [20] to allow for comparison over time. Choices in survey content were influenced by the Health Belief Model (e.g., inclusion of perceived susceptibility and severity to disease as well as perceived benefits of vaccinating) [24] and the Social Ecological Model (e.g., inclusion of social norms) [25].

### 2.3. Data Analyses

Our main analysis characterized those not yet up-to-date with COVID-19 vaccines, including their attitudes and values, trust in public health authorities, intentions to become up-to-date, and reasons for not yet doing so. We elucidated factors associated with being and intending to get up-to-date with COVID-19 vaccines. We also examined differences by race/ethnicity and looked for changes over time. Data were analyzed using Stata, version 16 [26].

A raking procedure was used to adjust design weights so that the sample was weighted to the population of adults at least 18 years old in the US. Black and Hispanic respondents were oversampled and down-weighted to reflect their proportion in the population. Further details on weighting are published elsewhere [19].

For the construct scale measuring trust in the CDC, a composite, linear score was generated (Supplemental Table S1). All answered items within the scale were coded (e.g., strongly disagree = 0, disagree = 1, agree = 2, strongly agree = 3, with negative items reversed) and summed to create the numerator, with the denominator being the total possible score (accounting for missing data). Thus, the scale ranged from 0 to 100 (0 being the least trust possible and 100 being the most trust possible) to facilitate comparisons across scales. Cronbach’s alpha coefficients for this scale were estimated to be 0.93, indicating strong reliability. The score was then dichotomized at the middle (50), creating “high trust” and “low trust” groups.

Univariate analyses (unweighted and weighted) were conducted for sociodemographic characteristics and vaccination/disease status. Otherwise, only weighted analyses were performed. Sociodemographic variables were cross-tabulated against being up-to-date on COVID-19 vaccines and intention to become up-to-date (among those not yet up-to-date) and stratified by sociodemographic characteristics of interest, such as race/ethnicity. Intention to vaccinate was reclassified as (1) definitely soon or probably soon (Likely), (2) probably eventually, only if required, or probably not (Uncertain), and (3) definitely not (Unlikely). Likert scale response options (strongly agree, agree, disagree, strongly disagree) were dichotomized to agree vs. disagree to facilitate straightforward analyses and interpretation. Other scale response options (e.g., very worried, somewhat worried, not very worried, not at all worried) were similarly dichotomized to reflect affirmative vs. negative (e.g., worried vs. not worried). Weighted proportions for responses to items assessed among the entire sample in multiple surveys were compared to assess changes over time.

For all weighted proportions, standard errors were estimated by Taylor-linearized variance estimation. For all cross-tabulations and comparisons over time, *p*-values were estimated by Pearson chi-squared proportion tests (significance level of α = 0.05). Bivariate odds ratios were estimated by generalized logistic binomial regression and a logit link function with binary dependent variables for being up-to-date on COVID-19 vaccines and intention to become up-to-date (among those not yet up-to-date), and binary independent variables for affirmative responses to dichotomized survey items (or dummy indicator variables for categorical survey items). All reported odds ratios are unadjusted.

## 3. Results

### 3.1. Study Population and Survey Weighting

The survey was fielded among 5323 Ipsos KnowledgePanel members and returned 2787 completed responses, of which 2561 qualified for the study (based on eligibility criteria and survey quotas) (Supplemental Figure S1). Sociodemographic characteristics and vaccination status of the study population (unweighted and weighted) are presented in Table 1. Weighting had a limited impact other than by race/ethnicity (with oversampling of Black and Hispanic populations), since the Ipsos KnowledgePanel was designed to represent the US adult population. Weighted data are generalizable to US adults.

### 3.2. Vaccination Status

Although 85% of the weighted sample reported receiving at least one dose of a COVID-19 vaccine, less than two-thirds (63%) reported being up-to-date on their COVID-19 vaccines (Table 2).

#### 3.2.1. Sociodemographic Characteristics Associated with Vaccination Status

Sociodemographic factors negatively associated with being up-to-date included younger age, less education, lower income, being a parent, living in the southern or midwestern regions of the US, living in a non-metro area, and identifying as Republican.

#### 3.2.2. Vaccine Attitudes Associated with Vaccination Status

Positive vaccine attitudes were positively associated with being up-to-date on COVID-19 vaccines, and negative vaccine attitudes were negatively associated. For example, the strongest association identified was among those who believed COVID-19 vaccines important in getting back to a normal life, who had nearly 16 times higher odds of being up-to-date compared to those who did not (odds ratio: 15.60; 95%CI: 11.84–20.55). Vaccinating against influenza during the past year was positively associated with being up-to-date on COVID-19 vaccines (OR: 6.99; 95%CI: 5.53–8.83), while having ever had the COVID-19 disease was negatively associated (OR: 0.57; 95%CI: 0.47–0.71). Perceived COVID-19 disease severity was not associated with being up-to-date.

#### 3.2.3. Trust in the CDC Associated with Vaccination Status

About two-thirds (68%) of the weighted sample reported high trust in the CDC according to our construct scale. Those with high trust in the CDC had nearly seven times higher odds of being up-to-date on COVID-19 vaccines compared to those with low trust (OR: 6.56; 95%CI: 5.18–8.32).

#### 3.2.4. Confidence in COVID-19 Information Sources Associated with Vaccination Status

Most (86%) of the weighted sample reported confidence in their doctor as a source of COVID-19 information. Nearly three-quarters reported confidence in their health department (74%), scientists/doctors from universities (73%), and the CDC (70%). Individual medical professionals also had the confidence of the majority, such as the Surgeon General (68%), CDC Director Dr. Rochelle Walensky (63%), NIAID, NIH Director Dr. Anthony Fauci (62%), and Former CDC Director and Surgeon General Dr. David Satcher (62%). About one-third reported confidence in the news (37%), their religious leader (31%), and trusted non-medical community members (31%). Only 13% reported confidence in COVID-19 information from social media.

Confidence in COVID-19 information from all sources we inquired about were positively associated with being up-to-date on COVID-19 vaccines, though associations were strongest with health professionals listed, such as one’s doctor (OR: 9.05; 95%CI: 6.23–13.16) and the CDC (OR: 9.05; 95%CI: 7.04–11.64), and weakest with those listed from other fields, such as religious leaders (OR: 1.45; 95%CI: 1.15–1.82) and trusted non-medical community members (OR: 1.59; 95%CI: 1.23–2.06).

### 3.3. Characterizing Those Not Up-to-Date on COVID-19 Vaccines

#### 3.3.1. Sociodemographic Characteristics among Those Not Up-to-Date

Most of those not up-to-date on their COVID-19 vaccines were under 45 years of age (58%), without a bachelor’s degree (76%), making under $75,000 annually (53%), and Republican or Independent (82%). About one-quarter (26%) of those not up-to-date had received a flu vaccine within the past year.

#### 3.3.2. Sociodemographic Characteristics Associated with Vaccine Intentions among Those Not Up-to-Date

Although only 12% of those not yet up-to-date indicated they were likely to get up-to-date as soon as possible and 42% were unlikely to ever get up-to-date, almost half (46%) were still uncertain (Table 3). Democrats not yet up-to-date had higher odds of being likely (OR: 25.94; 95%CI: 10.48–64.20) or uncertain (OR: 6.59; 95%CI: 3.84–11.31) vs. unlikely to get up-to-date than Republicans (Supplemental Table S2). Non-white Americans not yet up-to-date had higher odds of being likely or uncertain (vs. unlikely) to get up-to-date than white Americans; for example, both Black and Hispanic Americans had about five times higher odds of being likely (vs. unlikely) to get up-to-date than white Americans.

#### 3.3.3. Vaccine Attitudes Associated with Vaccine Intentions among Those Not Up-to-Date

Many of the vaccine attitudes associated with being up-to-date in Table 2 were also strongly associated with being likely or uncertain (vs. unlikely) to get up-to-date (Supplemental Table S2). In addition, those who expected the COVID-19 disease to be severe if they got it had nine times higher odds of being likely (vs. unlikely) to get up-to-date (OR: 9.19; 95%CI: 4.53–18.64).

#### 3.3.4. Information Sources Associated with Vaccine Intentions among Those Not Up-to-Date

Confidence in COVID-19 information from all sources we inquired about other than trusted non-medical community members were positively associated with being likely to get up-to-date on COVID-19 vaccines. Associations were strongest with the health professionals listed, such as Dr. Fauci (OR: 33.86; 95%CI: 15.91–72.07) and the CDC (OR: 32.64; 95%CI: 16.06–66.33).

#### 3.3.5. Reasons to Vaccinate among Those Uncertain about Getting Up-to-Date

More than half of those uncertain about getting up-to-date on COVID-19 vaccines reported believing that vaccines are important in stopping the spread of infection (59%) and helping the US get back to normal (53%) (Table 3). Fewer than half of the uncertain reported most or all of their family members (47%) and friends (39%) had gotten vaccinated against COVID-19, and that if their main doctor (46%) or a close family member (40%) or close friend (33%) recommended it, they would likely vaccinate. About one-fifth of the uncertain worried about spreading COVID-19 to their family (21%) or their friends, neighbors, and coworkers (28%). Only 12% of the uncertain thought COVID-19 would be severe if they got it. Although 62% of the uncertain already felt knowledgeable about COVID-19 vaccines, 31% wanted more information about them.

#### 3.3.6. Specific Vaccine Concerns among Those Uncertain about Getting Up-to-Date

The most common concerns about COVID-19 vaccines among those uncertain about getting up-to-date included their potential side effects that have not been figured out yet (88%), the speed of their development (77%), their newness (75%), the safety of their ingredients (69%), drug companies making a lot of money off of them (67%), allergic reactions to them (65%), and experimenting on people with them (63%). Other less common but still prevalent concerns among the uncertain included immediate side effects, such as aches/fatigue/fever (52%), some vaccines being made from aborted fetuses (38%), not enough diversity (“people of my race/ethnicity”) in vaccine studies (37%), potential changes to DNA (37%), and potential effects on fertility (33%).

#### 3.3.7. Other Reasons Not to Vaccinate among Those Uncertain about Getting Up-to-Date

About half of those uncertain about getting up-to-date reported worrying about the safety of COVID-19 vaccines (56%), thinking COVID-19 vaccines are not likely to protect against new variants (48%), and believing that the government is not acting in their best interest (45%). More than a third of the uncertain reported preferring to take other precautions (such as masking and social distancing) rather than getting a shot (42%), needing more time to learn and think more about it (41%), thinking they would not get very sick if they got COVID-19 (40%), not trusting pharmaceutical companies (38%), thinking vaccines unlikely to protect against either COVID-19 or “Long COVID” (37%), believing COVID-19 is no worse than the seasonal flu (36%), wanting to wait to see what happens to others who vaccinate (36%), and believing it better to develop immunity by getting sick rather than by getting a shot (33%). Fewer than one-fifth of the uncertain reported social media posts making them wary of vaccinating (18%), not thinking they were at risk of getting COVID-19 (16%), and their friends/family not wanting to vaccinate (15%). Only 6% reported not having access to vaccines, only 5% reported it too difficult to register for vaccination, and only 3% reported not knowing how to register.

#### 3.3.8. Information Sources among Those Uncertain about Getting Up-to-Date

Four-fifths (80%) of those uncertain about getting up-to-date were confident in their doctor as a source of COVID-19 information. Other health professionals listed had the confidence of about half of the uncertain, such as health departments (60%), university scientists and doctors (57%), the CDC (53%), the Surgeon General (52%), and Dr. Fauci (41%). About one-quarter of the uncertain were confident in those listed from other fields, such as religious leaders (25%) and trusted non-medical community members (24%). One-fifth (20%) of the uncertain trusted the news for COVID-19 information, and only 9% trusted social media.

### 3.4. Differences by Race and Ethnicity

#### 3.4.1. Vaccination Status

About the same proportion (62–64%) of white, Black, and Hispanic Americans reported being up-to-date on their COVID-19 vaccines (Table 4). Among those not yet up-to-date, about two-thirds of Black (68%) and Hispanic (70%) Americans remained open to getting up-to-date on COVID-19 vaccines (either likely to vaccinate or uncertain), compared to about half (51%) of white Americans (*p* < 0.01). In contrast, more white Americans (57%) were vaccinated against flu than Black and Hispanic Americans (46%) (*p* < 0.01).

#### 3.4.2. Reasons to Vaccinate

Black and especially Hispanic Americans also tended to report reasons to vaccinate more frequently than white Americans. More than three-quarters of Black (77% and 75%) and Hispanic (80% and 79%) Americans reported believing vaccines important in stopping the spread of infection and helping the US get back to normal, respectively, compared to about two-thirds of white Americans (68% and 66%) (*p* < 0.01). Nearly half of Hispanic Americans worried about spreading COVID-19 to their family (47%) or their friends, neighbors, and coworkers (43%), compared to about one-third of Black Americans (36% and 31%, respectively) and one-quarter of white Americans (24% and 21%, respectively) (*p* < 0.01). However, Black Americans were the least likely to report that most or all of their family members (59%) and friends (50%) had gotten vaccinated against COVID-19, compared to white (64% and 57%, respectively) and Hispanic (69% and 58%, respectively) Americans (*p* < 0.01). Four-fifths (80%) of white Americans already felt knowledgeable about COVID-19 vaccines, compared to three-quarters (75%) of Black and 63% of Hispanic Americans (*p* < 0.01). Only one-quarter (25%) of white Americans wanted more information about COVID-19 vaccines, compared to 40% of Black and 41% of Hispanic Americans (*p* < 0.01).

#### 3.4.3. Specific Vaccine Concerns and Other Reasons Not to Vaccinate

Among those not yet up-to-date, white Americans reported many of the specific concerns and other reasons not to vaccinate more frequently than Black and Hispanic Americans. White Americans were more likely to be concerned about the potential side effects of COVID-19 vaccines that have not been figured out yet (92%), the speed of their development (87%), their newness (83%), and drug companies making a lot of money off of them (80%), compared to Black Americans (83%, 76%, 68%, and 62%, respectively) and Hispanic Americans (77%, 68%, 65%, and 65%, respectively) (*p* < 0.01). White Americans were more likely to report not thinking they would get very sick if they got COVID-19 (48%), not believing COVID-19 to be any worse than the flu (55%), not thinking vaccines likely to protect them from COVID-19 (59%), new variants of COVID-19 (67%), or “Long COVID” (59%), and believing it better to develop immunity to COVID-19 by getting sick rather than getting a shot (55%), compared to Hispanic Americans (36%, 32%, 43%, 48%, and 39%, respectively) and Black Americans (24%, 23%, 40%, 46%, 43%, and 30%, respectively) (*p* < 0.01). White Americans were also more likely to report not trusting the pharmaceutical companies that have developed the vaccine (60%) and not believing that the government acts in their best interest (66%), compared to Hispanic Americans (43% and 45%, respectively) and Black Americans (43% and 49%, respectively) (*p* < 0.01).

However, white Americans were the least likely to report concerns regarding not enough diversity (“people of my race/ethnicity”) in vaccine studies (31%), followed by Hispanic (43%) and then Black (55%) Americans (*p* < 0.01). White Americans were also less likely to prefer taking other precautions (such as masking and social distancing) to getting a shot (35%), compared to Hispanic (51%) and Black (61%) Americans (*p* < 0.01). White Americans were the least likely to report not having access to vaccines (4%), not knowing how to register for vaccination (2%), and it being too difficult to register (2%), compared to Hispanic Americans (9%, 6%, and 9%, respectively) and Black Americans (12%, 7%, and 8%, respectively) (*p* < 0.01).

#### 3.4.4. Information Sources

No difference was found by race/ethnicity in one’s confidence in their doctor or in trusted non-medical community members as sources of COVID-19 information. However, all other information sources listed had lower confidence among white Americans as compared to Black and Hispanic Americans. This includes both health professionals, such as the CDC (65% vs. 75% vs. 79%, respectively) and Dr. Fauci (55% vs. 74% vs. 73%, respectively), as well as other sources, such as religious leaders (25% vs. 45% vs. 41%, respectively) and social media (8% vs. 20% vs. 24%, respectively) (*p* < 0.01).

### 3.5. Changes over Time

Response frequency to most items repeated in our July 2021 and September 2022 survey waves did not differ significantly (Table 5). However, several measures did change dramatically over time. The proportion of US adults ever knowingly having COVID-19 disease more than quadrupled, from 10% in July 2021 to 46% in September 2022 (*p* < 0.01). The proportion thinking COVID-19 would be severe if they got it decreased by 7%, from 23% to 15% (*p* = 0.02). At least 10% fewer respondents believed COVID-19 vaccines important in stopping the spread of infection and getting back to a normal life (*p* < 0.01). The proportion of respondents not yet up-to-date who reported social media posts making them wary of vaccinating decreased by 14% (*p* = 0.01). About one-quarter fewer of those not yet up-to-date reported needing more time to think (*p* = 0.02) or wanting to wait to see what happened to others before vaccinating (*p* < 0.01), or concerns about immediate side effects (*p* = 0.04) or allergic reactions (*p* = 0.01). Although knowing how to register for vaccination increased by 3% over this time (*p* < 0.01), access to vaccination decreased by 12% (*p* < 0.01).

## 4. Discussion

Although 85% of the weighted sample reported receiving at least one dose of a COVID-19 vaccine, fewer than two-thirds (63%) reported being up-to-date on their COVID-19 vaccines. These rates correspond to CDC data, which estimated that 87% of US adults had received at least one dose as of September 2022 [6], but are substantially higher than the rates reported by the Kaiser Family Foundation (KFF), which estimated that 77% of US adults had received at least one dose and 47% were up-to-date as of September 2022 [27].

The CDC now considers adults “up-to-date” if they have received at least one dose of the updated bivalent COVID-19 vaccine [28,29]. However, at the time of this survey (the beginning of September 2022), “up-to-date” was considered to mean fully vaccinated (with a primary series) and boosted, without specifying that the booster should be the most recent version, since the updated bivalent booster had just been authorized and was not yet widely available [12]. This makes the timing of our data on being “up-to-date” particularly interesting, as it captures the proportion of the US adult population having received a monovalent booster dose just prior to it becoming obsolete. Similarly, we report the intentions, concerns, and reasons for not vaccinating among those who had not yet received any booster by the time the updated bivalent boosters became available.

The CDC no longer reports vaccine coverage for those receiving any booster dose, specifying only those who have received the updated bivalent booster dose. As of 23 March 2023, only about one-fifth (20%) of US adults had received the updated booster and were, thus, “up-to-date”. This highlights the substantial decline in coverage with the bivalent booster compared to the initial monovalent booster.

COVID-19 vaccines will likely continue to be periodically updated to maintain protection against new variants that arise. We must, therefore, continue to regularly characterize those not “up-to-date” and strive to improve upon coverage with updated boosters to maximize protection and reduce suffering.

Although 42% of those not yet up-to-date in our September 2022 survey reported being unlikely to ever get up-to-date, 12% likely would, and 46% were uncertain. This indicates that the majority of those not up-to-date were still considering it at this time. This remained true even after the widespread introduction of the bivalent booster; National Immunization Survey Adult COVID Module (NIS-ACM) data from November and December of 2022 showed that among US adults who finished their COVID-19 primary series, 27% had gotten a bivalent booster, 39% had not yet gotten a bivalent booster but were open to it, 12% were uncertain about getting a bivalent booster, and 21% were reluctant to get a bivalent booster [30]. This demonstrates the continued need to reach those who are vaccinated but not yet up-to-date, the majority of whom have not ruled out receiving the updated booster and, thus, perhaps just need additional information, motivation, or convenience before doing so.

Similar to our previous surveys [21], data from this survey strongly associate being up-to-date on COVID-19 vaccines with sociodemographic characteristics, such as younger age, less education, lower income, being a parent, living in the southern or midwestern regions of the US, living in a non-metro area, and identifying as Republican. This suggests continued clustering of under-vaccinated persons both geographically and socially. Communities with low vaccine coverage have fueled outbreaks of other vaccine-preventable diseases, such as measles and pertussis, despite relatively high coverage nationally [31], and should be prioritized for vaccination campaigns.

Despite similar vaccine coverage by race/ethnicity, Hispanic and Black Americans not yet up-to-date were more likely than white Americans to remain open to getting up-to-date and report reasons to vaccinate, and less likely than white Americans to report most specific concerns and other reasons not to vaccinate. The most prevalent concerns among those not yet up-to-date on COVID-19 vaccines remained their newness and safety, as well as suspicion of pharmaceutical companies and government. More than half of those uncertain about getting up-to-date believed vaccines to be important to fighting the pandemic, but nearly half believed vaccines unlikely to protect them against new variants. Outreach and messaging should support decision-making among the uncertain by emphasizing the efficacy of updated boosters against currently circulating strains and addressing concerns in an empathetic and comprehensible manner.

Interestingly, perceived COVID-19 disease severity was not associated with being up-to-date. This may be related to perceived vaccine efficacy; that is, those up-to-date on their COVID-19 vaccines felt protected against COVID-19 disease and, thus, expected mild symptoms if they had a breakthrough case. We only measured perceived vaccine efficacy among those not yet up-to-date; however, we found it strongly associated with intentions to get up-to-date, which is a good proxy for being up-to-date.

Many vaccine attitudes did not change between our July 2021 and September 2022 survey waves, but those that did are likely interrelated. As COVID-19 disease prevalence increased, perceived disease severity decreased, which makes sense due to the corresponding increase in natural immunity after infection, as well as the increase in contagiousness but decrease in severity of the Omicron variant, which had emerged in November 2021 and quickly became the primary variant in circulation [32]. Although certain vaccine concerns decreased (such as immediate side effects and allergic reactions), the perceived importance of vaccination in ending the pandemic also decreased, likely due to the cumulative lived experience of knowing people who vaccinated safely yet seeing the pandemic prolonged in spite of vaccination. Worryingly, access to vaccination decreased by 12% in this timeframe. Access also remains inequitable; Black and Hispanic Americans were 2–4 times more likely than white Americans to report not having access to vaccines, not knowing how to register for vaccination, and finding it too difficult to register. Continued, consistent, easy, and equitable access to vaccination should be reprioritized to increase the likelihood of achieving high coverage with updated boosters.

Most (86%) of our survey respondents trusted their own doctor for COVID-19 information, even among those uncertain about getting up-to-date (80%), and without significant differences by race/ethnicity. This is reflected in the literature; doctors have been the most credible and frequently used source of vaccine information for a long time [33], and a recommendation from one’s doctor to vaccinate is a strong predictor of uptake for COVID-19 [21,34,35] and other vaccines [36,37]. Unvaccinated providers are far less likely to recommend vaccinating to their patients and harbor many of the same concerns as the public [35]. This reinforces the need to prioritize distributing updated resources to healthcare providers as new variants and boosters arise, to aid both in their own vaccine decision-making and in their efforts to support their patients’ decision-making.

The main limitation of this study is its cross-sectional nature; it provides a snapshot at a single point in time. However, the survey was well-timed to capture a critical time in the pandemic, measuring vaccination status immediately following authorization of the new bivalent boosters. We also made some interesting comparisons to one of our earlier surveys which also recruited participants from the Ipsos KnowledgePanel. Our data are subject to the limitations of self-reporting, and the estimates presented are unadjusted for contextual factors.

Some findings from this study may not be generalizable to countries other than the US. For example, studies have found higher income to be associated with increased vaccine hesitancy in other countries, such as Slovenia [38] and Australia [39], while our study found the opposite. Such divergences by country are likely due both to confounding effects within sociodemographic factors and differences in specific political climates between countries. The political polarization of COVID-19 vaccines in the US has greatly increased vaccine hesitancy among Republicans, who make up higher proportions of the country’s rural low-income populations. Thus, current associations between income and vaccine hesitancy in the US may be largely due to confounding by political affiliation, and the confounding effects of sociodemographic factors on each other may vary by country.

Despite much publicly available survey data assessing attitudes about COVID-19 vaccines, many analyses have not undergone peer review, and their quality varies (especially in terms of internal and external validity). This study is strengthened by the rigorous work put into our survey instrument and analyses, the quality of Ipsos KnowledgePanel as a well-established nationally representative panel, and the oversampling of Black and Hispanic populations to have power for more precise estimates when stratifying or comparing by race/ethnicity.

This precise characterization of vaccine intentions, attitudes, and values of those not yet up-to-date in September 2022 is meant to aid public health authorities at all levels in their efforts to support public vaccine decision-making through communication and outreach programs. Immunization programs should reprioritize convenient and free access to vaccines, especially in communities with low coverage. Messaging should emphasize the safety and efficacy of COVID-19 vaccines, explain how these vaccines were created so quickly, and remind people of the remaining threat of the disease. Messages may be more trusted coming from sources other than government and industry, such as local healthcare providers. Public health immunization programs must improve the uptake of updated boosters against new variants of COVID-19 to effectively control this evolving virus, with renewed efforts to support the decision-making of those uncertain about getting up-to-date.

## Figures and Tables

**Table 1:** Sociodemographic characteristics and vaccination status of the September 2022 survey sample: unweighted and weighted.

Sociodemographic Characteristics	Unweighted N (%)	Weighted % ^a^	Sociodemographic Characteristics	Unweighted N (%)	Weighted % ^a^
All	2561 (100)		Household Annual Income		
Gender			<$50 k	847 (33.1)	29.9
Female	1274 (49.7)	51.4	$50–75 k	452 (17.6)	16.4
Male	1287 (50.3)	48.6	$75–100 k	346 (13.5)	13.1
Age (years)			$100–150 k	413 (16.1)	17.8
18–29	308 (12)	19.9	$150 k+	503 (19.6)	22.8
30–44	679 (26.5)	25.7	Political Affiliation		
45–59	669 (26.1)	23.9	Republican	517 (20.2)	25.3
≥60	905 (35.3)	30.5	Democrat	998 (39.1)	32.8
Education			Independent/other	1039 (40.7)	41.9
<High School	223 (8.7)	9.6	Metropolitan Statistical Area Status		
High School	589 (23)	28.1	Non-metro	276 (10.8)	13.4
Some College	757 (29.6)	27.1	Metro	2285 (89.2)	86.6
Bachelor’s or Higher	576 (22.5)	19.6	Parent Status		
Master’s or Higher	416 (16.2)	15.5	No children < 18	1794 (70.1)	71.4
Race/Ethnicity			At least one child < 18	767 (29.9)	28.6
White, non-Hispanic	997 (38.9)	62.8	Influenza Vaccination ^b^		
Black, non-Hispanic	609 (23.8)	12.0	Not vaccinated	1144 (45.2)	46.1
Hispanic	838 (32.7)	16.9	Vaccinated	1386 (54.8)	53.9
Other, non-Hispanic	117 (4.6)	8.3	COVID-19 Vaccination		
Region			Not vaccinated	329 (13.3)	15.5
Northeast	415 (16.2)	17.3	Vaccinated (>1 dose)	2140 (86.7)	84.5
Midwest	455 (17.8)	20.7	COVID-19 Disease		
South	1036 (40.5)	38.2	Never had	1432 (56.7)	55.9
West	655 (25.6)	23.8	Ever had	1093 (43.3)	44.1

^a^ Weights were produced using iterative proportional fitting, so respondents were weighted to represent US adults; Black and Hispanic respondents were weighted to adjust for oversampling which was carried out to allow for sufficient power to perform analyses stratified by race/ethnicity. ^b^ Respondents reported influenza vaccination within the past 12 months; these data were collected just prior to the 2022–2023 influenza season, so reflect the 2021–2022 influenza season.

**Table 2:** Frequency and odds of being up-to-date on COVID-19 vaccinations by vaccine attitudes, trust in the CDC, and sociodemographic characteristics.

Survey Items	Total (%) ^a^	COVID Vaccines, % ^b^		Odds Ratio (95%CI) ^c^	*p*-Value ^d^
		Not Up-to-Date	Up-to-Date		
All	100	37	63		
Constructs ^e^					
Trust in the Centers for Disease Control and Prevention (CDC)	68	44	84	6.56 (5.18–8.32)	<0.01
Sociodemographic Characteristics					
Gender					0.78
Female	51	52	51	ref ^k^	
Male	49	48	49	1.03 (0.84–1.27)	
Age (years)					<0.01
18–29	20	28	15	ref ^k^	
30–44	26	30	23	1.40 (1.01–1.93)	
45–59	24	22	26	2.16 (1.54–3.02)	
60+	31	20	37	3.35 (2.41–4.66)	
Education (attained)					<0.01
<High school	10	13	7	ref ^k^	
High school	28	34	23	1.23 (0.83–1.81)	
Some college	27	29	26	1.60 (1.10–2.35)	
Bachelor’s or higher	20	16	22	2.46 (1.65–3.68)	
Master’s or higher	16	7	21	5.02 (3.20–7.88)	
Race/Ethnicity					0.02
White, non-Hispanic	63	66	61	ref ^k^	
Black, non-Hispanic	12	12	12	1.06 (0.82–1.36)	
Hispanic	17	17	17	1.11 (0.88–1.39)	
Other, non-Hispanic	8	6	10	1.89 (1.14–3.13)	
Region					<0.01
Northeast	17	14	19	ref ^k^	
Midwest	21	23	19	0.60 (0.43–0.85)	
South	38	45	34	0.55 (0.41–0.74)	
West	24	18	27	1.07 (0.77–1.51)	
Household income					<0.01
<$50 k	30	35	25	ref ^k^	
$50–75 k	16	18	16	1.23 (0.91–1.67)	
$75–100 k	13	14	13	1.39 (1.00–1.93)	
$100–150 k	18	18	18	1.44 (1.05–1.97)	
$150 k+	23	16	28	2.47 (1.82–3.36)	
Political affiliation					<0.01
Republican	25	39	17	ref ^k^	
Democrat	33	18	43	5.27 (3.97–6.99)	
Independent/other	42	43	40	2.07 (1.59–2.69)	
Metropolitan Statistical Area status (metro vs. non-metro)	87	81	90	2.09 (1.54–2.85)	<0.01
Parent status (at least one child < 18 vs. no children < 18)	29	36	24	0.57 (0.46–0.72)	<0.01
Affirmative Responses To Survey Items ^f^					
Vaccination and Disease Status					
Vaccinated against flu within the past year	54	26	72	6.99 (5.53–8.83)	<0.01
Ever knowingly had COVID disease	44	53	39	0.57 (0.47–0.71)	<0.01
Confidence in sources for information about COVID-19					
My doctor	86	73	96	9.05 (6.23–13.16)	<0.01
My local or state health department	74	50	90	8.99 (6.91–11.69)	<0.01
Scientists and doctors from the Centers for Disease Control and Prevention (CDC)	70	43	87	9.05 (7.04–11.64)	<0.01
The Surgeon General	68	42	86	8.29 (6.48–10.60)	<0.01
Scientists and doctors from universities	73	49	89	8.65 (6.65–11.26)	<0.01
Dr. Anthony Fauci from the National Institutes of Health	62	33	80	8.33 (6.58–10.55)	<0.01
Dr. Rochelle Walensky, CDC Director	63	35	81	8.02 (6.33–10.15)	<0.01
Dr. David Satcher, Morehouse School of Medicine, Former CDC Director and Surgeon General	62	35	80	7.39 (5.85–9.34)	<0.01
My religious leader	31	26	33	1.45 (1.15–1.82)	<0.01
Other non-medical people in my community that I trust (specify)	31	25	34	1.59 (1.23–2.06)	<0.01
What I see on the news	37	16	50	5.17 (4.04–6.61)	<0.01
What I see on social media (Facebook, Twitter, etc.)	13	9	15	1.78 (1.28–2.46)	<0.01
Agreement With Other COVID-19 Likert Scale Items (For Adults)					
I am worried I may accidentally spread COVID-19 to my family members in the next six months	30	19	37	2.56 (2.02–3.25)	<0.01
I am worried I may accidentally spread COVID-19 to my friends, neighbors, or co-workers in the next six months	27	17	33	2.37 (1.85–3.03)	<0.01
If I get COVID-19, I think it will be severe	14	13	14	1.18 (0.86–1.61)	0.30
COVID-19 vaccines are important to stopping the spread of infection in the US	72	44	91	12.73 (9.67–16.75)	<0.01
COVID-19 vaccines are important to helping the US get back to a normal life	71	40	91	15.60 (11.84–20.55)	<0.01
Most or all of my family members have gotten vaccinated against COVID-19	65	40	82	6.63 (5.25–8.36)	<0.01
Most or all of my friends have gotten vaccinated against COVID-19	58	33	75	6.14 (4.90–7.69)	<0.01
I feel knowledgeable about the COVID-19 vaccine	76	31	89	1.98 (1.56–2.52)	<0.01
I would like more information on COVID-19 vaccines	30	24	81	1.62 (1.29–2.05)	<0.01
Agreement With Other COVID-19 Likert Scale Items (for Children)					
COVID-19 can be a serious disease for some children	83	71	92	4.72 (3.54–6.28)	<0.01
I am concerned about the safety of COVID-19 vaccine in children	61	78	50	0.27 (0.21–0.34)	<0.01
Vaccinating children against COVID-19 is important to end the pandemic and get back to normal	65	33	84	11.01 (8.61–14.09)	<0.01
It is better for children to develop immunity to COVID-19 by getting sick rather than by getting a shot	37	65	19	0.13 (0.10–0.17)	<0.01
COVID-19 in children is no worse than a cold or the flu	42	64	28	0.22 (0.18–0.28)	<0.01
I would support a requirement for children to be vaccinated against COVID-19 to attend school	56	27	74	7.86 (6.21–9.95)	<0.01
My child(ren)’s doctor recommended that my child(ren) be vaccinated against COVID-19 once authorized by the FDA ^g^	67	46	88	8.91 (5.61–14.14)	<0.01
If not: I would feel more comfortable giving my child(ren) a COVID-19 vaccine if my child(ren)’s doctor recommended it ^g^	27	20	60	6.10 (2.58–14.38)	<0.01
I would feel more comfortable giving my child(ren) a COVID-19 vaccine that was fully approved for children by the FDA (instead of just authorized for emergency use) ^g^	66	51	80	3.94 (2.59–5.99)	<0.01
Agreement With General Vaccine Likert Scale Items					
I am confident in the safety of vaccines	79	60	93	8.58 (6.41–11.5)	<0.01
I do not trust a vaccine unless it has already been safely given to millions of other people	46	63	37	0.34 (0.27–0.42)	<0.01
I am concerned about some of the ingredients in vaccines	47	68	34	0.24 (0.20–0.31)	<0.01
Vaccine recommendations from the Centers for Disease Control and Prevention (CDC) are a good fit for me	72	48	88	8.27 (6.39–10.71)	<0.01
I am concerned that the government and drug companies experiment on people like me	41	64	27	0.21 (0.17–0.26)	<0.01
The benefits of vaccines are much bigger than their risks	78	58	93	9.73 (7.30–12.96)	<0.01

Red text indicates survey items assessing negative vaccine attitudes. ^a^ Column percentages (of total sample), weighted for national representativeness; ^b^ column percentages (of up-to-date/not up-to-date) (except for first row “All” which is a row percentage), weighted for national representativeness; ^c^ odds ratio (95% confidence interval) of being up-to-date vs. not up-to-date for affirmative survey response vs. not; ^d^ using the Pearson chi-square test (significance level of alpha = 5%); for non-dichotomous categorical variables, *p*-values for differences between all categories included in top row with variable name; ^e^ for the construct scale measuring trust in the CDC, a composite, linear score was generated. All answered items within the scale were coded (e.g., strongly disagree = 0, disagree = 1, agree = 2, strongly agree = 3; with negative items reversed) and summed to create the numerator, with the denominator being the total possible score (accounting for missing data). Thus, the scale ranged from 0 to 100 (0 being the least trust possible and 100 being most trust possible). The score was then dichotomized at the middle (50), creating “high trust” and “low trust” groups; ^f^ Likert scale response options (strongly agree, agree, disagree, strongly disagree) were dichotomized to agree/disagree, results for agreement shown; other scale response options were dichotomized to reflect affirmative/negative, results for affirmative shown; ^g^ asked only to respondents with children < 18; ^k^ reference value for logistic regression of categorical variables.

**Table 3:** Frequency and odds of intentions to get up-to-date on COVID-19 vaccinations by vaccine attitudes, trust in the CDC, and sociodemographic characteristics.

Survey Items	Total Not Up-to-Date (%) ^a^	COVID Vaccine Intentions, % ^b^			*p*-Value ^c^
		Likely	Uncertain	Unlikely	
All	100	12	46	42	
Constructs ^e^					
Trust in the Centers for Disease Control and Prevention (CDC)	44	84	56	15	<0.01
Sociodemographic Characteristics					
Gender					0.97
Female	52	52	51	52	
Male	48	48	49	48	
Age (years)					0.46
18–29	28	33	28	24	
30–44	30	32	32	29	
45–59	22	17	21	23	
60+	20	17	19	24	
Education (attained)					0.17
<High school	13	22	14	10	
High school	34	24	34	39	
Some college	29	28	29	29	
Bachelor’s or higher	16	19	16	14	
Master’s or higher	7	7	7	8	
Race/Ethnicity					<0.01
White, non-Hispanic	66	37	63	76	
Black, non-Hispanic	12	20	13	9	
Hispanic	17	31	18	12	
Other, non-Hispanic	6	11	7	3	
Region					0.14
Northeast	14	13	12	16	
Midwest	23	18	25	20	
South	45	50	40	48	
West	18	19	22	15	
Household income					0.68
<$50 k	35	43	38	35	
$50–75 k	18	18	17	17	
$75–100 k	14	9	15	11	
$100–150 k	18	16	15	20	
$150 k+	16	14	15	16	
Political affiliation					<0.01
Republican	39	15	31	52	
Democrat	18	42	22	5	
Independent/other	43	43	47	43	
Metropolitan Statistical Area status (metro vs. non-metro)	81	86	84	78	0.19
Parent status (at least one child < 18 vs. no children < 18)	36	48	34	33	0.06
Affirmative Responses To Survey Items ^f^					
Vaccination and Disease Status					
Vaccinated against flu within the past year	26	45	27	20	<0.01
Ever knowingly had COVID disease	53	49	49	55	0.31
Reasons to not get a COVID-19 vaccine ^h^					
I do not think I am at risk of getting COVID-19					<0.01
Yes	23	20	16	30	
No	52	62	49	54	
Do not know	25	18	35	16	
I do not think I would get very sick if I got COVID-19					<0.01
Yes	45	19	40	52	
No	33	53	33	30	
Do not know	21	28	27	18	
I do not believe COVID-19 is any worse than the seasonal flu					<0.01
Yes	47	15	36	64	
No	36	70	41	23	
Do not know	17	15	22	14	
I do not think the COVID-19 vaccines are likely to protect me from COVID-19					<0.01
Yes	54	19	37	78	
No	28	56	37	12	
Do not know	18	25	26	10	
I do not think the COVID-19 vaccines are likely to protect me from new variants of COVID-19					<0.01
Yes	61	26	48	79	
No	21	49	26	11	
Do not know	18	25	26	10	
I do not think the COVID-19 vaccines are likely to protect me from “Long COVID”					<0.01
Yes	53	22	37	74	
No	21	51	26	11	
Do not know	26	26	37	15	
It is better to develop immunity to COVID-19 by getting sick rather than by getting a shot					<0.01
Yes	50	14	33	76	
No	26	69	35	5	
Do not know	24	17	32	19	
I would rather take other precautions (such masking and social distancing) than get a shot					<0.01
Yes	41	20	42	42	
No	51	66	45	53	
Do not know	9	14	13	4	
I am worried about the safety of COVID-19 vaccines					<0.01
Yes	66	23	56	83	
No	25	67	32	10	
Do not know	9	10	13	7	
I do not like needles					0.03
Yes	23	35	24	21	
No	74	55	72	77	
Do not know	4	10	4	3	
I do not trust how the vaccine was developed					<0.01
Yes	57	17	41	81	
No	29	63	40	10	
Do not know	14	20	19	10	
I do not trust the pharmaceutical companies that have developed the vaccine					<0.01
Yes	54	19	38	79	
No	28	49	39	12	
Do not know	18	32	24	10	
I do not believe that the government is acting in my or my family’s best interest					<0.01
Yes	60	22	45	83	
No	23	52	32	7	
Do not know	17	26	23	10	
I do not have access to where vaccines are being given					0.04
Yes	5	10	6	4	
No	89	77	85	91	
Do not know	7	12	9	4	
I do not know how to register to get a vaccine					<0.01
Yes	3	13	3	3	
No	93	81	89	94	
Do not know	4	6	7	3	
I know how to register to get a vaccine but it is too difficult					0.03
Yes	4	11	5	2	
No	89	78	86	93	
Do not know	6	11	9	4	
My friends and/or family do not want to get the vaccine					<0.01
Yes	22	15	15	30	
No	62	74	68	53	
Do not know	16	12	17	17	
I have seen posts on social media that make me wary of the vaccine					<0.01
Yes	23	12	18	31	
No	69	72	72	63	
Do not know	8	16	10	6	
I want to wait to see what happens to others					<0.01
Yes	39	14	36	44	
No	51	70	49	51	
Do not know	10	16	15	5	
I need more time to learn and think more about it					<0.01
Yes	34	18	41	28	
No	59	64	49	68	
Do not know	7	18	10	3	
Other					0.02
Yes	25	15	19	32	
No	54	46	59	47	
Do not know	22	39	22	20	
Concerns regarding COVID-19 vaccines ^i^					
How fast COVID-19 vaccines were developed and made available to the public	84	58	77	89	<0.01
COVID-19 vaccines are new	79	37	75	85	<0.01
A lot of people who get the vaccine feel tired, achy, and get headaches and fever the next day	58	64	52	65	0.03
Some people have bad allergic reactions to COVID-19 vaccines	70	57	65	74	0.08
I am not sure the ingredients in COVID-19 vaccines are safe	78	59	69	87	<0.01
COVID-19 vaccines are made from mRNA	69	42	59	78	<0.01
COVID-19 vaccines might change my DNA	44	48	37	53	<0.01
COVID-19 vaccines might affect my fertility or ability to have children	41	52	33	46	0.01
There may be side effects to the COVID-19 vaccine that have not been figured out yet	91	67	88	92	<0.01
There were not enough people of my race/ethnicity who were a part of the vaccine studies	35	51	37	37	0.46
They are experimenting on people with the COVID-19 vaccine	73	59	63	84	<0.01
The drug companies are making a lot of money off of COVID-19 vaccines	75	63	67	83	<0.01
Some COVID-19 vaccines are made from aborted fetuses	48	57	38	58	<0.01
Confidence in sources for information about COVID-19					
My doctor	73	94	80	54	<0.01
My local or state health department	50	87	60	25	<0.01
Scientists and doctors from the Centers for Disease Control and Prevention (CDC)	43	87	53	17	<0.01
The Surgeon General	42	83	52	15	<0.01
Scientists and doctors from universities	49	88	57	26	<0.01
Dr. Anthony Fauci from the National Institutes of Health	33	78	41	10	<0.01
Dr. Rochelle Walensky, CDC Director	35	77	42	13	<0.01
Dr. David Satcher, Morehouse School of Medicine, Former CDC Director and Surgeon General	35	75	42	14	<0.01
My religious leader	26	40	25	24	0.02
Other non-medical people in my community that I trust (specify)	25	27	24	28	0.70
What I see on the news	16	44	20	5	<0.01
What I see on social media (Facebook, Twitter, etc.)	9	20	9	6	<0.01
Agreement With Other COVID-19 Likert Scale Items (For Adults)					
I am worried I may accidentally spread COVID-19 to my family members in the next six months	19	54	21	7	<0.01
I am worried I may accidentally spread COVID-19 to my friends, neighbors, or co-workers in the next six months	17	53	18	7	<0.01
If I get COVID-19, I think it will be severe	13	40	12	7	<0.01
COVID-19 vaccines are important to stopping the spread of infection in the US	44	97	59	10	<0.01
COVID-19 vaccines are important to helping the US get back to a normal life	40	94	53	9	<0.01
Most or all of my family members have gotten vaccinated against COVID-19	40	69	47	21	<0.01
Most or all of my friends have gotten vaccinated against COVID-19	33	54	39	16	<0.01
If my main doctor were to recommend that I take the COVID-19 vaccine, I would be likely to take it	28	96	46	6	<0.01
If a close family member were to recommend that I take the COVID-19 vaccine, I would be likely to take it	24	75	40	3	<0.01
If my close friends were to recommend that I take the COVID-19 vaccine, I would be likely to take it	20	73	33	3	<0.01
I feel knowledgeable about the COVID-19 vaccine	68	64	62	76	<0.01
I would like to get more information on COVID-19 vaccines	24	53	31	10	<0.01
Agreement With Other COVID-19 Likert Scale Items (for Children)					
COVID-19 can be a serious disease for some children	71	94	81	51	<0.01
I am concerned about the safety of COVID-19 vaccine in children	78	69	78	81	0.08
Vaccinating children against COVID-19 is important to end the pandemic and get back to normal	33	88	43	8	<0.01
It is better for children to develop immunity to COVID-19 by getting sick rather than by getting a shot	65	28	56	84	<0.01
COVID-19 in children is no worse than a cold or the flu	64	33	58	79	<0.01
I would support a requirement for children to be vaccinated against COVID-19 to attend school	27	83	31	5	<0.01
My child(ren)’s doctor recommended that my child(ren) be vaccinated against COVID-19 once authorized by the FDA ^g^	46	80	50	22	<0.01
If not: I would feel more comfortable giving my child(ren) a COVID-19 vaccine if my child(ren)’s doctor recommended it ^g^	20	81	28	6	<0.01
I would feel more comfortable giving my child(ren) a COVID-19 vaccine that was fully approved for children by the FDA (instead of just authorized for emergency use) ^g^	51	93	62	16	<0.01
Agreement With General Vaccine Likert Scale Items					
I am confident in the safety of vaccines	60	92	66	40	<0.01
I do not trust a vaccine unless it has already been safely given to millions of other people	63	50	63	64	0.08
I am concerned about some of the ingredients in vaccines	68	56	64	76	<0.01
Vaccine recommendations from the Centers for Disease Control and Prevention (CDC) are a good fit for me	48	92	58	22	<0.01
I am concerned that the government and drug companies experiment on people like me	64	51	57	76	<0.01
The benefits of vaccines are much bigger than their risks	58	87	64	36	<0.01

Red text indicates survey items assessing negative vaccine attitudes. ^a^ Column percentages (of respondents not up-to-date on COVID-19 vaccines), weighted for national representativeness; ^b^ column percentages (of corresponding intention categories) (except for first row “All” which is a row percentage), weighted for national representativeness; ^c^ using the Pearson chi-square test (significance level of alpha = 5%); for non-dichotomous categorical variables, *p*-values for differences between all categories included in top row with variable name; ^e^ construct scales combine scores for each relevant survey item (reversing negative items) and divide by maximum (e.g., 100 being complete trust and 0 being complete distrust); after dichotomizing at middle (50), binary variable represents high vs. low score (e.g., 1 being high trust and 0 being low trust); ^f^ Likert scale response options (strongly agree, agree, disagree, strongly disagree) were dichotomized to agree/disagree, results for agreement shown; other scale response options were dichotomized to reflect affirmative/negative, results for affirmative shown; ^g^ asked only to respondents with children < 18; ^h^ asked only to respondents not yet up-to-date on COVID-19 vaccines and not definitely planning to soon become up-to-date on COVID-19 vaccines; ^i^ asked only to respondents worried or uncertain about the safety of COVID-19 vaccines.

**Table 4:** Race/ethnicity by vaccine attitudes, trust in the CDC, and sociodemographic characteristics.

Survey Items	Total (%) ^a^	Race/Ethnicity (%) ^b^				*p*-Value ^c^
		White	Black	Hispanic	Other	
All	100	63	12	17	8	
Constructs ^d^						
Trust in the Centers for Disease Control and Prevention (CDC)	68	63	74	74	81	<0.01
Affirmative Responses To Survey Items ^e^						
Vaccination and Disease Status						
Vaccinated against flu within the past year	54	57	46	46	60	<0.01
Ever knowingly had COVID disease	44	44	34	48	50	0.01
Vaccinated against COVID (at least one dose)	85	83	84	87	95	<0.01
Up-to-date on COVID vaccines	63	62	63	64	75	0.02
If not up-to-date: intentions to get up-to-date						<0.01
Likely to soon get up-to-date	12	7	20	22	24	
Uncertain about getting up-to-date	46	44	49	48	54	
Unlikely to ever get up-to-date	42	49	32	30	22	
Reasons to not get a COVID-19 vaccine ^h^						
I do not think I am at risk of getting COVID-19						0.20
Yes	23	21	22	26	23	
No	52	56	48	38	52	
Do not know	25	23	30	36	25	
I do not think I would get very sick if I got COVID-19						<0.01
Yes	45	48	24	36	44	
No	33	31	38	33	48	
Do not know	21	21	38	31	8	
I do not believe COVID-19 is any worse than the seasonal flu						<0.01
Yes	47	55	23	32	32	
No	36	31	44	40	67	
Do not know	17	15	33	28	1	
I do not think the COVID-19 vaccines are likely to protect me from COVID-19						0.01
Yes	54	59	40	43	43	
No	28	24	32	31	50	
Do not know	18	17	27	26	8	
I do not think the COVID-19 vaccines are likely to protect me from new variants of COVID-19						<0.01
Yes	61	67	46	48	31	
No	21	16	29	24	53	
Do not know	18	16	25	28	16	
I do not think the COVID-19 vaccines are likely to protect me from “Long COVID”						<0.01
Yes	53	59	43	39	29	
No	21	16	27	27	52	
Do not know	26	25	30	35	19	
It is better to develop immunity to COVID-19 by getting sick rather than by getting a shot						<0.01
Yes	50	55	30	45	42	
No	26	22	29	25	44	
Do not know	24	23	41	30	13	
I would rather take other precautions (such as masking and social distancing) than get a shot						<0.01
Yes	41	35	61	51	31	<0.01
No	51	57	28	35	63	
Do not know	9	8	11	14	6	
I am worried about the safety of COVID-19 vaccines						0.19
Yes	66	68	63	56	70	
No	25	23	25	29	30	
Do not know	9	10	12	15	0	
I do not like needles						0.01
Yes	23	22	30	23	24	
No	74	75	61	69	75	
Do not know	4	2	8	9	1	
I do not trust how the vaccine was developed						0.01
Yes	57	62	49	45	48	
No	29	26	28	32	47	
Do not know	14	13	23	23	6	
I do not trust the pharmaceutical companies that have developed the vaccine						<0.01
Yes	54	60	43	43	43	
No	28	24	30	31	50	
Do not know	18	16	27	26	7	
I do not believe that the government is acting in my or my family’s best interest						<0.01
Yes	60	66	49	45	51	
No	23	19	25	28	38	
Do not know	17	15	27	27	11	
I do not have access to where vaccines are being given						<0.01
Yes	5	4	12	9	3	
No	89	91	74	77	97	
Do not know	7	6	14	14	0	
I do not know how to register to get a vaccine						0.01
Yes	3	2	7	6	3	
No	93	93	82	85	95	
Do not know	4	4	11	9	2	
I know how to register to get a vaccine but it is too difficult						<0.01
Yes	4	2	8	9	12	
No	89	91	81	81	85	
Do not know	6	7	11	10	2	
My friends and/or family do not want to get the vaccine						<0.01
Yes	22	23	21	16	21	
No	62	62	52	61	78	
Do not know	16	15	26	23	1	
I have seen posts on social media that make me wary of the vaccine						0.16
Yes	23	22	24	27	19	
No	69	69	63	61	81	
Do not know	8	9	13	12	0	
I want to wait to see what happens to others						0.10
Yes	39	38	33	39	41	
No	51	53	47	45	50	
Do not know	10	9	20	15	8	
I need more time to learn and think more about it						0.03
Yes	34	32	40	38	37	
No	59	62	51	48	62	
Do not know	7	6	9	14	1	
Other						0.03
Yes	25	29	17	15	10	
No	54	49	55	57	77	
Do not know	22	21	28	28	13	
Concerns regarding COVID-19 vaccines ^i^						
How fast COVID-19 vaccines were developed and made available to the public	84	87	76	68	90	0.01
COVID-19 vaccines are new	79	83	68	65	81	<0.01
A lot of people who get the vaccine feel tired, achy, and get headaches and fever the next day	58	59	64	57	56	0.79
Some people have bad allergic reactions to COVID-19 vaccines	70	70	74	66	73	0.76
I am not sure the ingredients in COVID-19 vaccines are safe	78	80	77	70	75	0.34
COVID-19 vaccines are made from mRNA	69	72	64	56	69	0.07
COVID-19 vaccines might change my DNA	44	44	54	51	39	0.31
COVID-19 vaccines might affect my fertility or ability to have children	41	41	41	43	31	0.69
There may be side effects to the COVID-19 vaccine that have not been figured out yet	91	92	83	77	100	<0.01
There were not enough people of my race/ethnicity who were a part of the vaccine studies	35	31	55	43	60	<0.01
They are experimenting on people with the COVID-19 vaccine	73	75	72	68	79	0.65
The drug companies are making a lot of money off of COVID-19 vaccines	75	80	62	65	76	<0.01
Some COVID-19 vaccines are made from aborted fetuses	48	52	42	48	44	0.44
Confidence in sources for information about COVID-19						
My doctor	86	86	82	88	89	0.25
My local or state health department	74	71	76	80	82	<0.01
Scientists and doctors from the Centers for Disease Control and Prevention (CDC)	70	65	75	79	81	<0.01
The Surgeon General	68	63	72	76	82	<0.01
Scientists and doctors from universities	73	69	73	81	85	<0.01
Dr. Anthony Fauci from the National Institutes of Health	62	55	74	73	77	<0.01
Dr. Rochelle Walensky, CDC Director	63	58	67	72	73	<0.01
Dr. David Satcher, Morehouse School of Medicine, Former CDC Director and Surgeon General	62	57	67	71	76	<0.01
My religious leader	31	25	45	41	29	<0.01
Other non-medical people in my community that I trust (specify)	31	31	31	34	24	0.29
What I see on the news	37	33	43	49	42	<0.01
What I see on social media (Facebook, Twitter, etc.)	13	8	20	24	15	<0.01
Agreement With Other COVID-19 Likert Scale Items (For Adults)						
I am worried I may accidentally spread COVID-19 to my family members in the next six months	30	24	36	47	38	<0.01
I am worried I may accidentally spread COVID-19 to my friends, neighbors, or co-workers in the next six months	27	21	31	43	36	<0.01
If I get COVID-19, I think it will be severe	14	12	16	18	16	0.05
COVID-19 vaccines are important to stopping the spread of infection in the US	72	68	77	80	84	<0.01
COVID-19 vaccines are important to helping the US get back to a normal life	71	66	75	79	89	<0.01
Most or all of my family members have gotten vaccinated against COVID-19	65	64	59	69	76	<0.01
Most or all of my friends have gotten vaccinated against COVID-19	58	57	50	58	78	<0.01
I feel knowledgeable about the COVID-19 vaccine	76	80	75	63	79	<0.01
I would like to get more information on COVID-19 vaccines	30	25	40	41	37	<0.01
Agreement With Other COVID-19 Likert Scale Items (for Children)						
COVID-19 can be a serious disease for some children	83	82	91	81	86	0.01
I am concerned about the safety of COVID-19 vaccine in children	61	59	70	67	51	<0.01
Vaccinating children against COVID-19 is important to end the pandemic and get back to normal	65	59	73	71	80	<0.01
It is better for children to develop immunity to COVID-19 by getting sick rather than by getting a shot	37	38	33	39	29	0.11
COVID-19 in children is no worse than a cold or the flu	42	45	32	40	41	0.01
I would support a requirement for children to be vaccinated against COVID-19 to attend school	56	50	65	64	72	<0.01
My child(ren)’s doctor recommended that my child(ren) be vaccinated against COVID-19 once authorized by the FDA ^f^	67	60	69	69	93	<0.01
If not: I would feel more comfortable giving my child(ren) a COVID-19 vaccine if my child(ren)’s doctor recommended it ^f^	27	28	22	23	35	0.83
I would feel more comfortable giving my child(ren) a COVID-19 vaccine that was fully approved for children by the FDA (instead of just authorized for emergency use) ^f^	66	59	68	76	72	0.01
Agreement With General Vaccine Likert Scale Items						
I am confident in the safety of vaccines	79	78	77	81	88	0.05
I do not trust a vaccine unless it has already been safely given to millions of other people	46	43	56	54	46	<0.01
I am concerned about some of the ingredients in vaccines	47	45	58	52	42	<0.01
Vaccine recommendations from the Centers for Disease Control and Prevention (CDC) are a good fit for me	72	70	74	76	84	<0.01
I am concerned that the government and drug companies experiment on people like me	41	36	56	52	41	<0.01
The benefits of vaccines are much bigger than their risks	78	77	76	80	90	0.01

Red text indicates survey items assessing negative vaccine attitudes. ^a^ Column percentages (of total sample), weighted for national representativeness; ^b^ column percentages (of race/ethnicity) (except for first row “All” which is a row percentage), weighted for national representativeness; all race/ethnicity categories are non-Hispanic except for Hispanic (i.e., participants of Hispanic ethnicity were considered Hispanic regardless of their race); ^c^ using the Pearson chi-square test (significance level of alpha = 5%); for non-dichotomous categorical variables, *p*-values for differences between all categories included in top row with variable name; ^d^ construct scales combine scores for each relevant survey item (reversing negative items) and divide by maximum (e.g., 100 being complete trust and 0 being complete distrust); after dichotomizing at middle (50), binary variable represents high vs. low score (e.g., 1 being high trust and 0 being low trust); ^e^ Likert scale response options (strongly agree, agree, disagree, strongly disagree) were dichotomized to agree/disagree, results for agreement shown; other scale response options were dichotomized to reflect affirmative/negative, results for affirmative shown; ^f^ asked only to respondents with children; ^h^ asked only to respondents not yet up-to-date on COVID-19 vaccines and not definitely planning to soon become up-to-date on COVID-19 vaccines; ^i^ asked only to respondents worried or uncertain about the safety of COVID-19 vaccines.

**Table 5:** Changes in vaccine attitudes between July 2021 and September 2022.

Survey Items	July 2021 % (n = 2525) ^a^	Sept 2022 % (n = 2546) ^a^	% Dif	*p*-Value ^b^
Affirmative Responses To Survey Items ^c^				
Vaccination and Disease Status				
Vaccinated against flu within the past year	55.5	51.0	−4.5	0.64
Ever knowingly had COVID disease	9.9	45.6	35.6	<0.01
Reasons to not get a COVID-19 vaccine ^d^				
I do not think I am at risk of getting COVID-19	36.3	19.1	−17.2	0.22
I do not think I would get very sick if I got COVID-19	33.4	35.3	1.9	0.27
I do not believe COVID-19 is any worse than the seasonal flu	36.8	33.9	−2.9	0.11
I do not think the COVID-19 vaccines are likely to protect me from COVID-19	49.6	42.9	−6.8	0.23
I am worried about the safety of COVID-19 vaccines	86.7	68.3	−18.4	0.06
I do not like needles	25.8	27.0	1.2	0.76
I do not trust how the vaccine was developed	71.0	59.9	−11.0	0.27
I do not trust the pharmaceutical companies that have developed the vaccine	56.1	54.6	−1.5	0.99
I do not believe that the government is acting in my or my family’s best interest	68.4	57.2	−11.2	0.62
I do not have access to where vaccines are being given	1.3	13.6	12.3	<0.01
I do not know how to register to get a vaccine	13.4	10.1	−3.4	<0.01
I know how to register to get a vaccine but it is too difficult	6.7	5.0	−1.7	0.79
My friends and/or family do not want to get the vaccine	26.9	15.1	−11.8	0.39
I have seen posts on social media that make me wary of the vaccine	46.4	32.7	−13.6	0.01
I want to wait to see what happens to others	70.0	45.2	−24.9	<0.01
I need more time to learn and think more about it	60.3	38.4	−21.9	0.02
Concerns regarding COVID-19 vaccines ^e^				
How fast COVID-19 vaccines were developed and made available to the public	93.7	82.9	−10.7	0.23
COVID-19 vaccines are new	92.2	75.6	−16.7	0.12
A lot of people who get the vaccine feel tired, achy, and get headaches and fever the next day	82.5	57.8	−24.7	0.04
Some people have bad allergic reactions to COVID-19 vaccines	93.4	62.2	−31.2	0.01
I am not sure the ingredients in COVID-19 vaccines are safe	93.4	74.1	−19.2	0.10
COVID-19 vaccines are made from mRNA	83.3	54.9	−28.3	0.02
COVID-19 vaccines might change my DNA	54.1	41.9	−12.2	0.57
COVID-19 vaccines might affect my fertility or ability to have children	54.7	42.9	−11.8	0.31
There may be side effects to the COVID-19 vaccine that have not been figured out yet	98.2	86.8	−11.5	0.08
There were not enough people of my race/ethnicity who were a part of the vaccine studies	35.3	37.2	1.9	0.97
They are experimenting on people with the COVID-19 vaccine	80.3	72.2	−8.1	0.68
The drug companies are making a lot of money off of COVID-19 vaccines	73.1	74.8	1.7	0.97
Some COVID-19 vaccines are made from aborted fetuses	62.9	51.9	−10.9	0.38
Confidence in sources for information about COVID-19				
My doctor	81.5	85.4	3.8	0.31
My local or state health department	72.9	74.2	1.4	0.95
Scientists and doctors from the Centers for Disease Control and Prevention (CDC)	72.4	69.4	−3.0	0.80
The Surgeon General	68.1	68.5	0.4	0.99
Scientists and doctors from universities	75.3	71.9	−3.3	0.79
Dr. Anthony Fauci from the National Institutes of Health	61.3	63.7	2.4	0.84
Dr. Rochelle Walensky, CDC Director	64.7	61.1	−3.6	0.78
Dr. David Satcher, Morehouse School of Medicine, Former CDC Director and Surgeon General	64.7	61.8	−2.9	0.80
My religious leader	39.2	30.5	−8.7	0.09
Other non-medical people in my community that I trust (specify)	31.7	34.7	2.9	0.52
What I see on the news	42.9	33.0	−10.0	0.14
What I see on social media (Facebook, Twitter, etc.)	12.0	11.2	−0.8	0.63
Agreement With Other COVID-19 Likert Scale Items (For Adults)				
I am worried I may accidentally spread COVID-19 to my family members in the next six months	25.1	30.1	5.0	0.38
I am worried I may accidentally spread COVID-19 to my friends, neighbors, or co-workers in the next six months	21.7	27.1	5.4	0.34
If I get COVID-19, I think it will be severe	22.6	15.3	−7.3	0.02
COVID-19 vaccines are important to stopping the spread of infection in the US	87.5	77.0	−10.4	<0.01
COVID-19 vaccines are important to helping the US get back to a normal life	87.4	75.7	−11.6	<0.01
Most or all of my family members have gotten vaccinated against COVID-19	61.2	66.7	5.5	0.57
Most or all of my friends have gotten vaccinated against COVID-19	60.9	61.6	0.8	0.45
Agreement With General Vaccine Likert Scale Items				
I am confident in the safety of vaccines	84.4	81.7	−2.7	0.21
I do not trust a vaccine unless it has already been safely given to millions of other people	41.8	41.8	−0.1	0.21
I am concerned about some of the ingredients in vaccines	44.7	44.0	−0.7	0.53
Vaccine recommendations from the Centers for Disease Control and Prevention (CDC) are a good fit for me	79.4	72.3	−7.1	0.16
I am concerned that the government and drug companies experiment on people like me	42.1	39.2	−2.9	0.78
The benefits of vaccines are much bigger than their risks	84.3	80.1	−4.3	0.18

Red text indicates survey items assessing negative vaccine attitudes. ^a^ Column percentages (of corresponding sample), weighted for national representativeness; ^b^ using the Pearson chi-square test (significance level of alpha = 5%); ^c^ Likert scale response options (strongly agree, agree, disagree, strongly disagree) were dichotomized to agree/disagree, results for agreement shown; other scale response options were dichotomized to reflect affirmative/negative, results for affirmative shown; ^d^ asked only to respondents not yet up-to-date on COVID-19 vaccines and not definitely planning to soon become up-to-date on COVID-19 vaccines; ^e^ asked only to respondents worried or uncertain about the safety of COVID-19 vaccines.

## Data Availability

Deidentified individual participant data will not be made available.

## Supplementary Materials

The following supporting information can be downloaded at: https://www.mdpi.com/article/10.3390/jcm12123932/s1, Table S1: Composition and properties of construct scales, Table S2: Odds ratios for intentions to get up-to-date on COVID-19 vaccinations by vaccine attitudes, trust in the CDC, and sociodemographic characteristics, Figure S1: Inclusion and exclusion of participants.
