# Supplementary material for: Attitudes and Values of US Adults Not Yet Up-to-Date on COVID-19 Vaccines in September 2022
Source: J Clin Med. 2023 Jun 8;12(12):3932. doi: 10.3390/jcm12123932 (PMC10299362; doi:10.3390/jcm12123932)
Supplement: Supplementary file 1 [file jcm-12-03932-s001.zip › jcm-2408142_Table S1.pdf]

Table S1. Composition and Properties of Construct Scales.

In the first column, the name of each scale is bolded and the survey items contributing to each scale are listed below it. Numbers in the "Weighted (%)" columns indicate the percentage of the total weighted sample providing the response in that column header to the survey item in each row. The median and inter-quartile range (IQR) describe the distribution of the scale scores, and the Cronbach Alpha describes the reliability of the scale (values greater than 0.80 are generally considered to have good reliability, and the closer to 1.0 the better).

Scale Items [a]	Weighted (%)				Median (IQR) [b]	Cronbach Alpha (Covariance) [c]
	Strongly Agree	Agree	Disagree	Strongly Disagree		
**Trust in the Centers for Disease Control and Prevention (CDC)**					62 (48–69)	0.93 (0.31)
They do everything they should to protect the health of the population.	14	49	28	9		
They are partly responsible for the illegal drug problems in this country. [a]	8	23	48	20		
They recommend things for the public that aren't helpful. [a]	9	31	48	11		
They use resources well.	9	53	29	9		
They waste money on health problems. [a]	8	23	51	18		
They keep trying the same things to help the public, even when they don't work very well. [a]	10	41	42	6		
They come up with new ideas to solve health problems.	11	60	23	5		
They base recommendation on the best available science.	24	51	18	7		
They accurately inform the public of both health risks and benefits of medicines.	13	51	26	9		
They believe in what they recommend for the public.	19	57	19	5		
They quickly help the public with health problems.	10	51	31	8		
They are concerned about all people, without caring about who has more or less money.	20	48	23	9		
They are more concerned about some racial and ethnic groups than other groups. [a]	8	25	51	16		
They ensure the public is protected against diseases.	15	58	21	6		

[a] Responses to 4-point Likert scale items used as the basis for composite scales centralized around the middle options of "agree" and disagree" compared to "strongly agree" and "strongly disagree." Response options were scored and summed to create linear scores: strongly agree = 3, agree = 2, disagree = 1, strongly disagree = 0. Selected items ([a]) were reversed: strongly agree = 0, agree = 1, disagree = 2, strongly disagree = 3. These scores were then divided by the maximum to create a

scale with possible scores from 0 to 100 (e.g., 100 being complete trust and 0 being complete distrust). These continuous scores were also dichotomized at the middle (50) to allow for logistic analyses. [b] IQR: Inter Quartile Range. [c] Cronbach's alpha is a measure of internal consistency. Scales with Cronbach alpha values greater than 0.80 are generally considered to have good reliability.
