# Supplementary material for: Attitudes and Values of US Adults Not Yet Up-to-Date on COVID-19 Vaccines in September 2022
Source: J Clin Med. 2023 Jun 8;12(12):3932. doi: 10.3390/jcm12123932 (PMC10299362; doi:10.3390/jcm12123932)
Supplement: Supplementary file 1 [file jcm-12-03932-s001.zip › jcm-2408142_Table S2.pdf]

Table S2. Odds Ratios for Intentions to Get Up-to-date on COVID-19 Vaccinations by Vaccine Attitudes, Trust in CDC, and Sociodemographic Characteristics.

Survey Items	OR (95%CI) [d] Likely vs. Unlikely	Uncertain vs. Unlikely
ALL		
CONSTRUCTS [e]		
Trust in the Centers for Disease Control and Prevention (CDC)	29.67 (13.40–65.69)	7.07 (4.62–10.83)
SOCIODEMOGRAPHIC CHARACTERISTICS		
Gender		
Female	ref [k]	
Male	1.02 (0.61–1.71)	1.04 (0.73–1.48)
Age (years)		
18–29	ref [k]	
30–44	0.81 (0.40–1.62)	0.94 (0.57–1.55)
45–59	0.56 (0.25–1.25)	0.81 (0.47–1.38)
60+	0.52 (0.24–1.12)	0.66 (0.39–1.13)
Education (attained)		
<High School	ref [k]	
High School	0.28 (0.12–0.65)	0.65 (0.35–1.20)
Some College	0.45 (0.20–1.00)	0.74 (0.40–1.37)
Bachelors or Higher	0.64 (0.27–1.53)	0.87 (0.44–1.70)
Masters or Higher	0.41 (0.14–1.25)	0.63 (0.29–1.37)
Race/Ethnicity		
White, non-Hispanic	ref [k]	
Black, non-Hispanic	4.61 (2.41–8.79)	1.72 (1.11–2.66)
Hispanic	5.40 (2.92–9.97)	1.77 (1.19–2.63)
Other, non-Hispanic	7.89 (2.41–25.90)	2.74 (1.08–6.95)
Region		
Northeast	ref [k]	
Midwest	1.13 (0.47–2.73)	1.59 (0.90–2.82)
South	1.30 (0.62–2.75)	1.08 (0.64–1.80)

West	1.58 (0.65–3.79)	1.91 (1.06–3.45)
Household income		
<$ 50 k	ref [k]	
$ 50–75 k	0.84 (0.40–1.77)	0.92 (0.55–1.52)
$ 75–100 k	0.68 (0.28–1.67)	1.22 (0.71–2.08)
$ 100–150 k	0.64 (0.30–1.35)	0.67 (0.40–1.14)
$ 150 k+	0.70 (0.31–1.56)	0.87 (0.52–1.48)
Political affiliation		
Republican	ref [k]	
Democrat	25.94 (10.48–64.20)	6.59 (3.84–11.31)
Independent/Other	3.42 (1.45–8.05)	1.78 (1.21–2.62)
Metropolitan Statistical Area status (metro vs. non-metro)	1.77 (0.81–3.83)	1.42 (0.89–2.27)
Parent status (at least one child <18 vs. no children <18)	1.88 (1.11–3.18)	1.07 (0.74–1.55)
Affirmative Responses to Survey Items [f]		
Vaccination and Disease Status		
Vaccinated against flu within the past year	3.33 (1.90–5.85)	1.47 (0.96–2.25)
Ever knowingly had COVID disease	0.76 (0.45–1.28)	0.78 (0.54–1.11)
Reasons to not get a COVID-19 vaccine [h]		
I don’t think I am at risk of getting COVID-19		
Yes	ref [k]	ref [k]
No	1.79 (0.79–4.09)	1.68 (1.04–2.72)
Don't Know	1.78 (0.69–4.58)	4.08 (2.26–7.34)
I don’t think I would get very sick if I got COVID-19		
Yes	ref [k]	ref [k]
No	4.73 (2.03–11.01)	1.43 (0.92–2.22)
Don't Know	4.06 (1.68–9.83)	1.88 (1.15–3.07)
I do not believe COVID-19 is any worse than the seasonal flu		
Yes	ref [k]	ref [k]
No	12.82 (5.32–30.89)	3.17 (2.05–4.89)
Don't Know	4.47 (1.65–12.09)	2.85 (1.70–4.79)

I don’t think the COVID-19 vaccines are likely to protect me from COVID-19		
Yes	ref [k]	ref [k]
No	20.19 (8.56–47.60)	6.91 (4.16–11.46)
Don't Know	10.72 (3.95–29.10)	5.60 (3.31–9.47)
I don’t think the COVID-19 vaccines are likely to protect me from new variants of COVID-19		
Yes	ref [k]	ref [k]
No	13.6 (5.64–32.79)	3.98 (2.35–6.72)
Don't Know	7.55 (2.85–20.01)	4.30 (2.51–7.36)
I don’t think the COVID-19 vaccines are likely to protect me from “Long COVID”		
Yes	ref [k]	ref [k]
No	15.41 (6.04–39.31)	4.62 (2.70–7.93)
Don't Know	5.88 (2.21–15.65)	4.99 (3.11–7.98)
It is better to develop immunity to COVID-19 by getting sick rather than by getting a shot		
Yes	ref [k]	ref [k]
No	65.8 (23.41–184.95)	14.56 (7.49–28.34)
Don't Know	4.67 (1.73–12.60)	3.87 (2.45–6.12)
I’d rather take other precautions (like masking and social distancing) than get a shot		
Yes	ref [k]	ref [k]
No	2.63 (1.24–5.58)	0.87 (0.59–1.28)
Don't Know	6.69 (2.04–21.88)	3.07 (1.36–6.92)
I am worried about the safety of COVID-19 vaccines		
Yes	ref [k]	ref [k]
No	24.14 (10.88–53.52)	4.71 (2.78–7.96)
Don't Know	5.26 (1.75–15.74)	2.68 (1.41–5.09)
I do not like needles		
Yes	ref [k]	ref [k]
No	0.43 (0.20–0.94)	0.80 (0.50–1.26)
Don't Know	2.36 (0.68–8.15)	1.29 (0.46–3.63)
I do not trust how the vaccine was developed		

Yes	ref [k]	ref [k]
No	30.85 (13.16–72.33)	8.27 (4.91–13.94)
Don't Know	9.62 (3.59–25.78)	3.92 (2.24–6.87)
I do not trust the pharmaceutical companies that have developed the vaccine		
Yes	ref [k]	ref [k]
No	17.64 (7.59–41.03)	6.92 (4.27–11.24)
Don't Know	13.58 (5.28–34.95)	5.00 (2.88–8.66)
I do not believe that the government is acting in my or my family's best interest		
Yes	ref [k]	ref [k]
No	29.67 (11.43–76.97)	8.75 (4.93–15.53)
Don't Know	9.72 (3.70–25.50)	4.20 (2.45–7.17)
I do not have access to where vaccines are being given		
Yes	ref [k]	ref [k]
No	0.36 (0.12–1.02)	0.71 (0.29–1.75)
Don't Know	1.23 (0.31–4.83)	1.70 (0.53–5.45)
I do not know how to register to get a vaccine		
Yes	ref [k]	ref [k]
No	0.17 (0.05–0.56)	0.79 (0.26–2.44)
Don't Know	0.33 (0.07–1.63)	1.77 (0.43–7.35)
I know how to register to get a vaccine but it is too difficult		
Yes	ref [k]	ref [k]
No	0.19 (0.04–0.86)	0.45 (0.14–1.44)
Don't Know	0.57 (0.09–3.72)	1.07 (0.27–4.26)
My friends and/or family do not want to get the vaccine		
Yes	ref [k]	ref [k]
No	2.90 (1.19–7.07)	2.55 (1.59–4.08)
Don't Know	1.40 (0.45–4.36)	1.95 (1.08–3.51)
I have seen posts on social media that make me wary of the vaccine		
Yes	ref [k]	ref [k]
No	2.86 (1.13–7.21)	1.95 (1.27–2.99)
Don't Know	6.09	2.73

	(1.79–20.74)	(1.26–5.90)
I want to wait to see what happens to others		
Yes	ref [k]	ref [k]
No	4.29 (1.81–10.16)	1.17 (0.79–1.72)
Don't Know	9.85 (2.96–32.82)	3.62 (1.70–7.72)
I need more time to learn and think more about it		
Yes	ref [k]	ref [k]
No	1.48 (0.71–3.08)	0.49 (0.33–0.73)
Don't Know	8.62 (2.82–26.40)	2.03 (0.87–4.74)
Other		
Yes	ref [k]	ref [k]
No	2.16 (0.72–6.53)	2.11 (1.20–3.73)
Don't Know	4.22 (1.26–14.17)	1.85 (0.91–3.78)
Concerns regarding COVID-19 vaccines [i]		
How fast COVID-19 vaccines were developed and made available to the public	0.17 (0.06–0.46)	0.42 (0.25–0.71)
COVID-19 vaccines are new	0.10 (0.04–0.25)	0.52 (0.32–0.86)
A lot of people who get the vaccine feel tired, achy and get headaches and fever the next day	0.98 (0.36–2.65)	0.61 (0.41–0.90)
Some people have bad allergic reactions to COVID-19 vaccines	0.47 (0.18–1.25)	0.66 (0.43–1.02)
I am not sure the ingredients in COVID-19 vaccines are safe	0.22 (0.08–0.59)	0.34 (0.20–0.56)
COVID-19 vaccines are made from mRNA	0.20 (0.08–0.51)	0.41 (0.26–0.63)
COVID-19 vaccines might change my DNA	0.81 (0.33–2.00)	0.53 (0.36–0.79)
COVID-19 vaccines might affect my fertility or ability to have children	1.25 (0.50–3.09)	0.59 (0.39–0.88)
There may be side effects to the COVID-19 vaccine that haven’t been figured out yet	0.17 (0.06–0.51)	0.64 (0.35–1.19)
There were not enough people of my race/ethnicity who were a part of the vaccine studies	1.81 (0.73–4.49)	1.00 (0.67–1.49)
They are experimenting on people with the COVID-19 vaccine	0.28 (0.11–0.73)	0.33 (0.21–0.53)
The drug companies are making a lot of money off of COVID-19 vaccines	0.34 (0.13–0.9)	0.41 (0.26–0.64)
Some COVID-19 vaccines are made from aborted fetuses	0.97 (0.39–2.42)	0.45 (0.30–0.67)
Confidence in sources for information about COVID-19		
My doctor	13.08	3.28

	(5.78–29.59)	(2.22–4.83)
My local or state health department	19.56 (10.09–37.89)	4.40 (3.01–6.44)
Scientists and doctors from the Centers for Disease Control and Prevention (CDC)	32.64 (16.06–66.33)	5.36 (3.58–8.04)
The Surgeon General	27.61 (13.61–55.99)	6.00 (3.93–9.17)
Scientists and doctors from Universities	20.50 (9.83–42.74)	3.88 (2.66–5.67)
Dr. Anthony Fauci from the National Institutes of Health	33.86 (15.91–72.07)	6.69 (4.02–11.14)
Dr. Rochelle Walensky, Director of the Centers for Disease Control and Prevention	22.58 (11.31–45.05)	4.97 (3.14–7.88)
Dr. David Satcher, Morehouse School of Medicine, Former CDC Director and Surgeon General	18.45 (9.51–35.79)	4.49 (2.88–7.01)
My religious leader	2.11 (1.22–3.64)	1.04 (0.69–1.57)
Other non-medical people in my community that I trust (specify)	0.95 (0.53–1.70)	0.84 (0.54–1.31)
What I see on the news	14.35 (7.19–28.64)	4.56 (2.49–8.35)
What I see on social media (facebook, twitter, etc.)	3.73 (1.76–7.91)	1.52 (0.80–2.86)
Agreement With Other COVID-19 Likert Scale Items (For Adults)		
I am worried I may accidentally spread COVID-19 to my family members in the next six months	14.81 (7.88–27.81)	3.33 (1.99–5.60)
I am worried I may accidentally spread COVID-19 to my friends, neighbors, or co-workers in the next six months	14.16 (7.55–26.56)	2.79 (1.64–4.75)
If I get COVID-19, I think it will be severe	9.19 (4.53–18.64)	1.87 (0.97–3.58)
COVID-19 vaccines are important to stopping the spread of infection in the US	332.48 (126.73–872.24)	13.05 (8.01–21.26)
COVID-19 vaccines are important to helping the US get back to a normal life	147.39 (61.40–353.84)	11.28 (6.90–18.44)
Most or all of my family members have gotten vaccinated against COVID-19	8.13 (4.63–14.28)	3.27 (2.22–4.82)
Most or all of my friends have gotten vaccinated against COVID-19	6.26 (3.58–10.95)	3.39 (2.25–5.11)
If my main doctor were to recommend that I take the COVID-19 vaccine, I'd be likely to take it	1.08 (0.98–1.18)	1.00 (0.89–1.12)
If a close family member were to recommend that I take the COVID-19 vaccine, I'd be likely to take it	112.69 (36.04–352.35)	24.39 (10.25–58.04)
If my close friends were to recommend that I take the COVID-19 vaccine, I'd be likely to take it	91.27 (31.29–266.29)	16.23 (7.12–36.95)
I feel knowledgeable about the COVID-19 vaccine	0.55 (0.31–0.97)	0.51 (0.34–0.76)
I'd like to get more information on COVID-19 vaccines	10.47	4.22

	(5.70–19.24)	(2.59–6.88)
Agreement With Other COVID-19 Likert Scale Items (for Children)		
COVID-19 can be a serious disease for some children	14.37 (5.83–35.41)	4.04 (2.73–5.96)
I am concerned about the safety of COVID-19 vaccine in children	0.51 (0.29–0.91)	0.83 (0.53–1.28)
Vaccinating children against COVID-19 is important to end the pandemic and get back to normal	92.89 (39.27–219.75)	9.24 (5.41–15.79)
It is better for children to develop immunity to COVID-19 by getting sick rather than by getting a shot	0.07 (0.04–0.13)	0.24 (0.16–0.36)
COVID-19 in children is no worse than a cold or the flu	0.13 (0.07–0.23)	0.37 (0.25–0.54)
I would support a requirement for children to be vaccinated against COVID-19 to attend school	84.10 (40.67–173.94)	7.95 (4.45–14.20)
My child(ren)'s doctor recommended that my child(ren) be vaccinated against COVID-19 once authorized by the FDA [g]	14.18 (4.78–42.12)	3.50 (1.84–6.64)
If not: I would feel more comfortable giving my child(ren) a COVID-19 vaccine if my child(ren)'s doctor recommended it [g]	68.62 (10.69–440.7)	6.19 (1.79–21.37)
I would feel more comfortable giving my child(ren) a COVID-19 vaccine that was fully approved for children by the FDA (instead of just authorized for emergency use) [g]	69.65 (19.83–244.65)	8.34 (4.09–17.00)
Agreement With General Vaccine Likert Scale Items		
I am confident in the safety of vaccines	16.02 (7.55–33.99)	2.82 (1.95–4.06)
I do not trust a vaccine unless it has already been safely given to millions of other people	0.56 (0.33–0.95)	0.98 (0.68–1.41)
I am concerned about some of the ingredients in vaccines	0.4 (0.23–0.70)	0.56 (0.38–0.84)
Vaccine recommendations from the Centers for Disease Control and Prevention (CDC) are a good fit for me	40.79 (18.9–88.03)	4.98 (3.32–7.45)
I am concerned that the government and drug companies experiment on people like me	0.34 (0.20–0.59)	0.43 (0.29–0.64)
The benefits of vaccines are much bigger than their risks	12.03 (5.99–24.17)	3.09 (2.13–4.47)

Red text indicates survey items assessing negative vaccine attitudes. [a] Column percentages (of respondents not up-to-date on COVID-19 vaccines), weighted for national representativeness. [b] Column percentages (of corresponding intention categories) (except for first row "All" which is a row percentage), weighted for national representativeness. [c] using the Pearson chi-square test (significance level of alpha = 5%); bold indicates statistical significance ($p < 0.05$); for non-dichotomous categorical variables, p-values for differences between all categories included in top row with variable name. [d] Odds Ratio (95% Confidence Interval) of likeliness vs. unlikeliness or uncertainty vs. unlikeliness to receive COVID-19 vaccine for affirmative survey response vs. not; bold indicates statistical significance ($p < 0.05$) using the Pearson chi-square test (significance level of alpha = 5%). [e] Construct scales combine scores for each relevant survey item (reversing negative items) and divide by maximum (e.g., 100 being complete trust and 0 being complete distrust); after dichotomizing at middle (50), binary variable represents high vs low score (e.g., 1 being high trust and 0 being low trust). [f] Likert scale response options (strongly agree, agree, disagree, strongly disagree) were dichotomized to agree/disagree, results for agreement shown; other scale response options were dichotomized to reflect affirmative/negative, results for affirmative shown. [g] asked only to

respondents with children <18. [h] asked only to respondents not yet up-to-date on COVID-19 vaccines and not definitely planning to soon become up-to-date on COVID-19 vaccines. [i] asked only to respondents worried or uncertain about the safety of COVID-19 vaccines. [k] Reference value for logistic regression of categorical variables.
