# Supplementary figures and images for: Attitudes and Values of US Adults Not Yet Up-to-Date on COVID-19 Vaccines in September 2022
Source: J Clin Med. 2023 Jun 8;12(12):3932. doi: 10.3390/jcm12123932 (PMC10299362; doi:10.3390/jcm12123932)

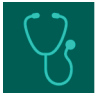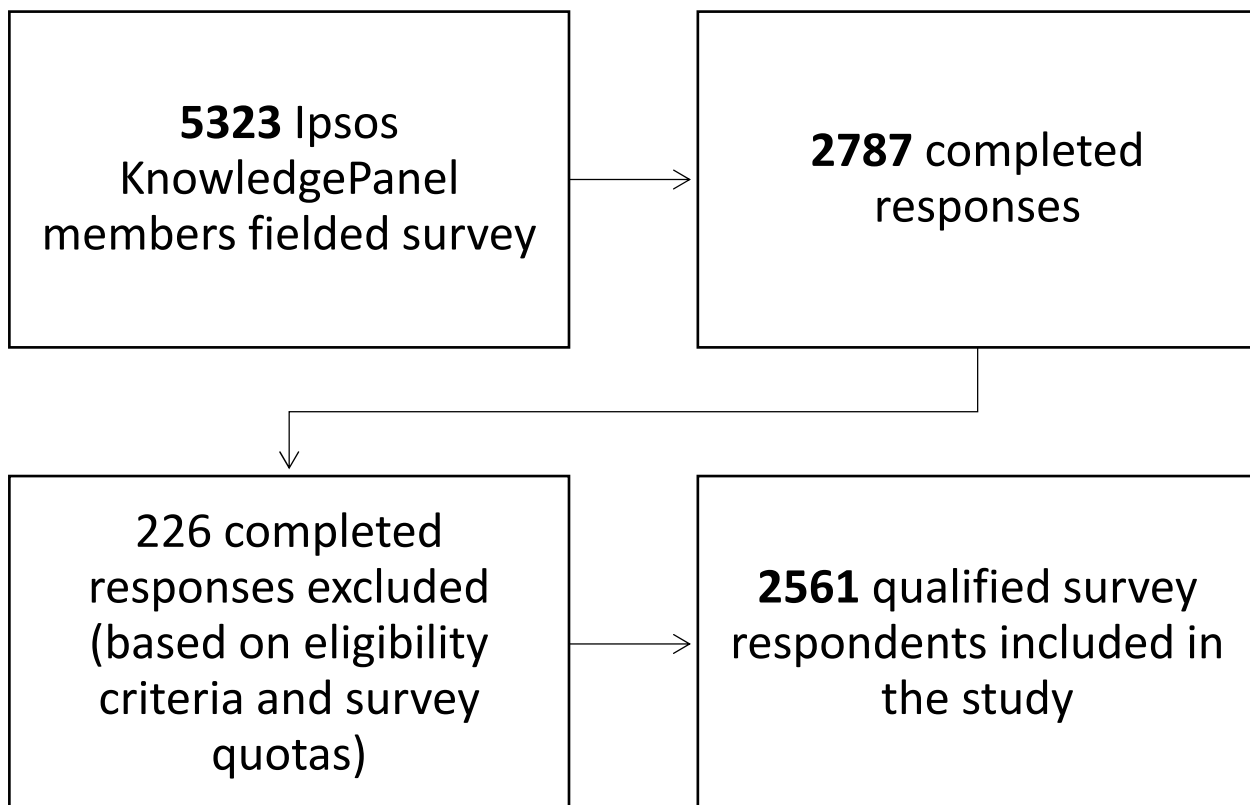

Figure S1. Inclusion and Exclusion of Participants.

Supplement: Supplementary file 1 [file jcm-12-03932-s001.zip › jcm-2408142_Figure S1.pdf]
